# Intervention planning and modification of the BUMP intervention: a digital intervention for the early detection of raised blood pressure in pregnancy

**DOI:** 10.1186/s40814-019-0537-z

**Published:** 2019-12-20

**Authors:** Rebecca Band, Lisa Hinton, Katherine L. Tucker, Lucy C. Chappell, Carole Crawford, Marloes Franssen, Sheila Greenfield, James Hodgkinson, Christine McCourt, Richard J. McManus, Jane Sandall, Mauro Dala Santos, Carmelo Velardo, Lucy Yardley

**Affiliations:** 1 Academic unit of psychology, University of Southampton, Southampton, SO17 1BJ UK; 2 Nuffield Department of Primary Care Health Sciences, Radcliffe Infirmary Quarter University of Oxford, Oxford, OX2 6GG UK; 3 Division of Women and Children’s Health, King’s College London, London, SE1 7EH UK; 4 Institute of Applied Health, University of Birmingham, Birmingham, B15 2TT UK; 5 Centre for Maternal and Child Health, School of Health Sciences, City University, London, EC1R IUW UK; 6 Institute of Biomedical Engineering, Department of Engineering Science, Building, University of Oxford, Oxford, OX3 7DQ UK; 7 School of Psychological Science, University of Bristol, Bristol, UK

**Keywords:** Hypertension, Pregnancy, Pre-eclampsia, Digital intervention, Intervention planning, Person-based approach

## Abstract

**Background:**

Hypertensive disorders in pregnancy, particularly pre-eclampsia, pose a substantial health risk for both maternal and foetal outcomes. The BUMP (Blood Pressure Self-Monitoring in Pregnancy) interventions are being tested in a trial. They aim to facilitate the early detection of raised blood pressure through self-monitoring. This article outlines how the self-monitoring interventions in the BUMP trial were developed and modified using the person-based approach to promote engagement and adherence.

**Methods:**

Key behavioural challenges associated with blood pressure self-monitoring in pregnancy were identified through synthesising qualitative pilot data and existing evidence, which informed guiding principles for the development process. Social cognitive theory was identified as an appropriate theoretical framework. A testable logic model was developed to illustrate the hypothesised processes of change associated with the intervention. Iterative qualitative feedback from women and staff informed modifications to the participant materials.

**Results:**

The evidence synthesis suggested women face challenges integrating self-monitoring into their lives and that adherence is challenging at certain time points in pregnancy (for example, starting maternity leave). Intervention modification included strategies to address adherence but also focussed on modifying outcome expectancies, by providing messages explaining pre-eclampsia and outlining the potential benefits of self-monitoring.

**Conclusions:**

With an in-depth understanding of the target population, several methods and approaches to plan and develop interventions specifically relevant to pregnant women were successfully integrated, to address barriers to behaviour change while ensuring they are easy to engage with, persuasive and acceptable.

## Introduction

Hypertension affects approximately 10% of women during pregnancy and may be an indication of pre-eclampsia, when arising around or after 20 weeks gestation [1]. Hypertensive disorders and pre-eclampsia are associated with adverse maternal and foetal outcomes [2, 3], with diagnosis ordinarily made during antenatal appointments. One potential way to improve detection is to ask women to self-monitor their own blood pressure at home throughout the second half of pregnancy to facilitate early detection of rising blood pressure (BP) in the absence of symptoms between appointments [4]. The literature on Self-monitoring BP (SMBP) in the general population suggests it can provide accurate estimates on which to base clinical decisions, is easy to incorporate into daily routines and facilitates patient understanding of self-management [5–9]. While this literature largely relates to an older, non-pregnant population, there is emerging evidence to suggest that SMBP may also be beneficial in pregnancy [10, 11].

## Background to the trials

### The BUMP trials (BUMP1 and BUMP2)

The BUMP (Blood Pressure Self-Monitoring in Pregnancy) programme of work has been developed to trial at-risk pregnancy clinical protocols using self-monitoring of blood pressure and includes two linked trials that aim to investigate whether BP self-monitoring in pregnancy improves the detection of raised BP during higher risk pregnancies (BUMP1) and whether self-monitoring reduces systolic BP during hypertensive pregnancy (BUMP2). Based on current literature, these will be the largest randomised controlled trials of blood pressure self-monitoring in pregnancy completed to date. Both use telemonitoring interventions. The primary outcome of the BUMP1 trial will be time to detection of pregnancy hypertension compared with usual antenatal care. This article describes the development activities undertaken for BUMP1. BUMP2 followed a parallel approach; further information may be requested from the authors. There will be an integral qualitative and quantitative process evaluation in both the BUMP1 and 2 trials [12]. The BUMP trials recruited participants from secondary care maternity units across the UK. BUMP1 aimed to recruit a minimum of 2262 pregnant women at higher risk of pregnancy hypertension and BUMP2 aimed to recruit a minimum of 512 pregnant women with either gestational or chronic hypertension. The BUMP1 primary outcome is the time to the first recording of raised BP by a healthcare professional. The BUMP2 primary outcome is mean systolic BP between baseline and delivery recorded by healthcare professionals. Other outcomes will include maternal and perinatal outcomes, quality of life and adverse events.

### BUMP key target behaviours

The following key behaviours in participating women are targeted by the BUMP telemonitoring system:

- Self-monitoring BP (SMBP) once a day, at least three times per week from 20 weeks’ gestation until delivery
- Submission of BP readings to the automated telemonitoring service via the BUMP app or SMS service. The telemonitoring service provides automated feedback regarding the BP readings submitted (for example acknowledging normal readings and requesting action for very high or very low readings)
- Responding to feedback messages from the telemonitoring system (i.e. taking additional BP readings or seeking support based on those readings, as advised and appropriate) when their BP is outside of the normal range

### The BUMP pilot

The BUMP pilot was a prospective cohort study, which aimed to facilitate early detection of pre-eclampsia [13]. A sample of 201 pregnant women identified as being at higher risk for pre-eclampsia (as defined by the NICE guidance) were recruited between 12- and 16-week gestation and asked to take morning and evening home blood pressure readings on 3 days per week for the duration of their pregnancy, starting from approximately 20 weeks until 6 weeks post-partum. Readings were recorded in a diary but could also be sent via SMS to a telehealth system [4]. A traffic light system was used for participants to interpret readings, with associated actions for low- or above-target readings. A small embedded qualitative study suggested that SMBP was acceptable and might help women feel both reassured and empowered [8]. However, the pilot data revealed persistence with SMBP reduced as pregnancy progressed, suggesting that further development work was necessary to ensure adherence would be maintained throughout pregnancy before testing in two linked trials [4] (ref protocol paper).

The pilot study highlighted several behavioural challenges to be addressed before the main trials, specifically, that further work was necessary to maintain long-term adherence to SMBP particularly through periods of transition (e.g. finishing work and starting maternity leave) and stress [8]. As the population at higher risk of pre-eclampsia comprises around half of all pregnant women [8, 11, 13], a second key design feature emerged: the BUMP1 intervention materials needed to be designed in an accessible way to promote engagement with a diverse group of women.

### Intervention planning and development

There has been much debate in recent years about greater clarity in reporting the development and content of complex behaviour change interventions [14, 15]. This paper therefore presents the intervention planning and development processes undertaken in preparation for the full-scale BUMP1 randomised controlled trial (RCT). This work took place in the first year of the programme grant in 2016 before recruitment to the full trial started in 2017. The development team included clinicians and researchers specialising in hypertension and obstetrics, experts in behaviour change, biomedical engineers with expertise in digital health and a social scientist with expertise in maternal health.

The person-based approach (PBA) to intervention development and planning, which has been successfully employed in other populations and trials [16, 17], was used. The PBA aims to elicit an in-depth understanding of the target user and their psychosocial context to guide the selection of key behavioural techniques in the specific context of the intervention, combined iteratively with evidence and relevant behavioural theory [18]. The perspectives of potential users are incorporated throughout to help intervention developers decide what are the most important features or aspects to focus on and how best to implement them [18]. This is achieved through in-depth qualitative work with target users (or the synthesis of existing qualitative literature, where it exists) and the development of “guiding principles” which outline the ways in which the intervention will meet the context-specific behavioural issues [18].

### Aims and objectives

i) Identify key behavioural issues, needs and challenges of self-monitoring during pregnancy, which includes developing guiding principles and selecting psychological theory to inform intervention planning and development
ii) To develop and refine participant materials to be used in the BUMP studies which address the key behavioural issues needs and challenges identified in part (i) using guiding principles and psychological theory
iii) To develop a logic model outlining the proposed mechanisms of change of the BUMP studies

## Methods

The methods outline the intervention planning methodology used to develop the intervention materials and theoretical modelling using the person-based approach to intervention planning [18]. The development process focused upon the ways in which behavioural content could be added to participant materials to increase participant adherence to the intervention (SMBP throughout pregnancy), and presented in a way which was appropriate for all women (regardless of the level of health literacy).

### Intervention planning methodology

**Fig. 1:**
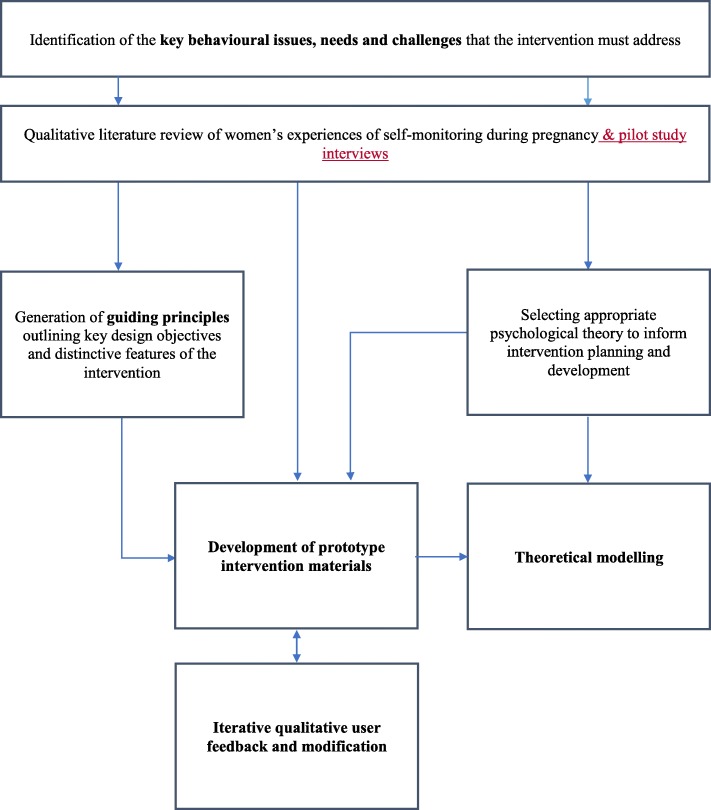
The intervention planning activities undertaken in the development of BUMP behavioural content

There were several interlinked intervention planning activities that were undertaken using the person-based approach to promote the key target behaviours outlined above. These included (i) identifying key behavioural issues, (ii) developing guiding principles, (iii) incorporating psychological theory and finally (iv) theoretical modelling. Figure 1 provides an overview of the connections between activities, which will be described in greater detail below before detailing how these informed the intervention development in BUMP1.

#### Identifying key behavioural issues, needs and challenges of self-monitoring during pregnancy

A secondary analysis of the qualitative interview data from the BUMP pilot study was undertaken [8]; this re-analysis enabled us to identify evidence for specific barriers and facilitators linked to the key target behaviours outlined above.

We could not identify further existing published evidence reporting women’s experiences of SMBP during pregnancy, so a non-exhaustive scoping search identified literature in related areas for potentially valuable insights. This consisted of general monitoring in pregnancy (*n* = 13) [19–30]; the use of pregnancy digital interventions (apps) (*n* = 6) [31–35]; women’s experiences of pre-eclampsia (*n* = 7) [36–42]; and SMBP in the general population (*n* = 8) [7, 9, 43–48]. We undertook a rapid review to ensure any existing evidence could be quickly incorporated into the planning process and inform design decisions [16]. Data extraction comprised a description of the facilitators and barriers (where relevant) and other findings reported within the papers, in addition to key considerations for the design of BUMP1 materials. Four key themes emerged from the extracted data relating to women’s understanding of the role of BP and the challenges they may face when engaging in SMBP in pregnancy (outlined in Table 1). Specifically, these highlighted a lack of knowledge about pre-eclampsia, difficulties understanding relevant health information, understanding the potential benefits of self-monitoring, and strategies to incorporate SMBP into everyday life.

**Table 1:** Key behavioural challenges facing women self-monitoring blood pressure in pregnancy

Key themes	Detail from the literature
Lack of knowledge about the risks of pre-eclampsia	Some women reported being unaware of the symptoms of pre-eclampsia and why it was an important health concern for themselves and their baby.
	Some women also did not understand why they were classified as being higher risk for pre-eclampsia.
	Women who developed pre-eclampsia had difficulty understanding why it had developed, particularly without accompanying symptoms or feeling ‘ill’.
Difficulties in understanding health information	Some women felt that information relating to raised blood pressure and pre-eclampsia was sometimes too technical for them to understand (i.e. medical terminology).
	Inconsistencies in health information were stressful or distressing.
	Women reported wanting to receive more information about their health status, presented in a way that is simple but comprehensive.
Understanding the benefit of self-monitoring	Some women felt that understanding the importance of SMBP helped them to engage with monitoring.
	Some women felt that understanding SMBP helped them to have confidence and feel empowered and reassured about their health status.
Women need help with strategies to fit SMBP into their daily lives	Women needed some flexibility with the monitoring schedule to allow them to incorporate it into their lives to suit them.
	Some women found the SMBP became difficult in the third trimester, especially when there were disruptions in daily life routines (such as finishing work), which worsened after the baby was born.
	Some women experienced guilt when they missed BP readings, which prevented them from reengaging with the intervention.

#### Guiding principles

Guiding principles allow for easy referral to the intervention design objectives and features required to meet the key behavioural challenges, when making design decisions related to the intervention in the development process [18]. For BUMP1, guiding principles focused primarily upon the ways in which the behavioural content could be used to motivate participants to engage in extended adherence to the intervention. Using the insights gained from identifying the key behavioural needs and challenges, a second priority was identified to ensure that all patient-facing information was presented in a way which was appropriate for all women (regardless of the level of health literacy). Key intervention features were outlined to ensure that each of the design objectives was met. The BUMP1 guiding principles are outlined in Table 2.

**Table 2:** BUMP guiding principles (for the person-based approach intervention development)

Design objective	Key intervention features
Design objective 1: to motivate participants to undertake long-term adherence to SMBP during pregnancy	Inform women of the benefits and safety of SMBP in pregnancy as a way to protect the health of themselves and their baby
	Emphasis on how to incorporate SMBP with daily routines, including promoting self-efficacy for overcoming potential barriers (i.e. during times of transition)
	Motivational text messages to be sent each week covering areas related to known barriers and facilitators
	Motivational messages reiterated in participant paper materials (such as participant booklet) to ensure all women receive the information
Design objective 2: participant materials are simple, clear and appropriate for women with lower health literacy	Short sentences, avoiding complex language and terminology (checked for appropriate reading levels)
	Visual representation of processes where possible to accompany the text
	All participant materials piloted with a diverse group of women and refined to ensure they are accessible and comprehensible to women with lower health literacy

#### Incorporating psychological theory to inform intervention planning and development

The behavioural synthesis of women’s experiences of SMBP during pregnancy identified that appropriate beliefs about pre-eclampsia and the benefits of self-monitoring are important barriers and facilitators of this target behaviour. In addition, factors promoting women’s self-efficacy, that is, women’s beliefs about their capability to successfully self-monitor, were important. These included factors such as having the necessary skills or confidence to reintegrate monitoring following setbacks [8]. Self-efficacy and outcome expectancies, that is, the likely outcomes people expect to occur as a result of the target behaviour, are central to social cognitive theory (SCT) [49]. This was therefore selected as an appropriate theory to guide the intervention planning and development process. SCT proposes that behaviour is the result of interactions between personal, behavioural and environmental factors [49]. The subsequent development of BUMP1 materials therefore focused on facilitating positive perceived outcomes of SMBP (i.e. the target behaviour) to promote the health of the women themselves and their baby (i.e. beneficial outcome expectancies), and were used to address the key behavioural issues (outlined in Table 1). In line with the PBA approach, autonomy-supportive language was used throughout (that is, careful use of language to promote a sense of autonomy over SMBP) which is essential in developing intrinsic motivation [50].

### Developing and refining participant materials using the PBA to ensure acceptability

As we developed the BUMP1 system and participant materials, we obtained iterative feedback on all materials. We included in this process pregnant women, new mothers and women with previous experience of pre-eclampsia (*n* = 19) via one focus group, one patient and public involvement (PPI) group and nine individual think-aloud interviews. Pregnant women were opportunistically approached by a research nurse, and those who agreed were sent the latest version of the study documents by RB. ‘Think aloud’ interviews were conducted by RB, where the participant read the information and gave reactions over the telephone, ensuring the content was understandable. RB and LH both attended PPI groups linked to two London hospitals to obtain group feedback on the materials. The feedback was collated, and if there was anything unappealing or might result in disengagement this was noted for discussion. This was an iterative process with minor changes made where necessary before the materials were given to the next participants. The development team (which included clinicians and researchers specialising in hypertension and obstetrics, experts in behaviour change, biomedical engineers with expertise in digital health and a social scientist with expertise in maternal health) also provided feedback on suggested changes at each iteration. The results for each of these activities (text messages, participant booklet and PIS) are presented below.

### Theoretical modelling

In line with the MRC best practice guidance [51], a BUMP1 logic model was developed to provide a testable, visual representation of the hypothesised mechanisms of behaviour change. This brought together the intervention planning activities and draws on SCT to illustrate the hypothesised relationships between modified outcome expectancies, increased self-efficacy and self-monitoring, and how these are anticipated to improve early detection of pre-eclampsia. The key behavioural components within the BUMP interventions were increasing participant motivation for self-monitoring to avoid potential harm to themselves and their baby, introducing self-monitoring (including instruction on how and when to monitor for optimal accuracy), and factors aimed at increasing engagement and adherence (such as ongoing reminders and providing women with strategies to overcome setbacks). Key behaviour change techniques (BCTs), as coded using the 93-item version (V1) behaviour change taxonomy [14], were linked to each intervention component. Classifying the intervention using standardised terminology (such as BCTs) promotes clarity around intervention ingredients.

## Results

### Developing the BUMP1 system

**Fig. 2:**
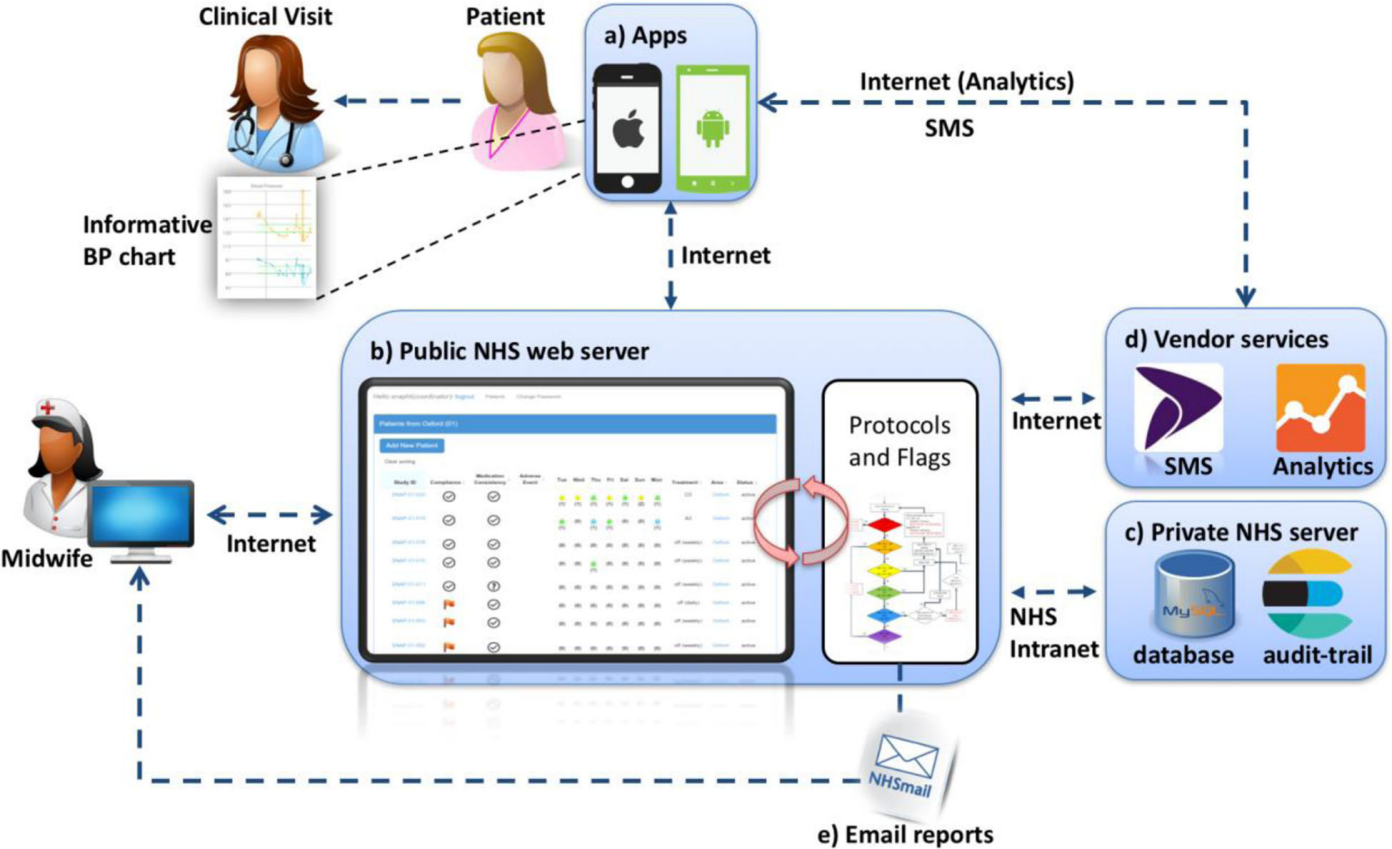
Illustration of the BUMP telemonitoring system software and network architecture. The SMS and the BUMP apps (**a**) can be used. The latter has an informative blood pressure (BP) chart that can be used during a clinical visit. The web application (**b**) receives the BP readings, and a rule-based algorithm assigns the BP level and suggests the next action to the user. The clinical and audit-trail data (**c**) are stored in a database server within the NHS intranet. Specialised vendor services, such as the Esendex SMS service and the Google Analytics (**d**), are used to enable the SMS service and anonymised website usage data collection, respectively. A weekly email report of abnormal readings or missing data issues (**e**), which can also be visualised as Flags on the website, is sent to authorised midwives

The BUMP1 system includes a smartphone app (Android and iOS) and a SMS-based communication service. The main system components are illustrated in Fig. 2. It was implemented using multi-platform web technologies and communication standards and was deployed via an NHS-managed server, behind the firewall of the Oxford University Hospitals NHS Foundation. Functionality testing was undertaken by the development team to test different combinations of normal and abnormal BP readings and user behaviours (e.g. poor adherence or numerous, unrequested readings) over a prolonged period followed by user testing with pregnant women. Access to the webpages of the BUMP1 application was designed via secure login to the participants, their clinicians and the research team. Trial participants submit their SMBP readings via the BUMP1 app or SMS service and in return, they receive reminders and automatic responses according to a rule-based algorithm developed with the clinical team. The system requests participants to make contact with clinicians in the case of high or low readings, and confirms normal readings where appropriate. Participants can switch between the app and the SMS service, for example where a mobile phone signal will not support internet connections but is good enough for texts.

### BUMP1 participant materials

All pre-existing participant materials (from the BUMP pilot) were edited using the PBA in order to address the second guiding principle (Table 2) and ensure that intervention materials were acceptable to women regardless of the level of health literacy. The language and instructions were simplified across all documents to ensure they were as easy to follow as possible. In addition, the information was condensed to avoid repetition and confusion; several documents were combined into one participant booklet with a view to helping ensure that women felt able to trust the information provided. Key behavioural messages were also incorporated where possible (e.g. reassuring women that an occasionally missed reading would not matter as long as they took their BP as soon as possible). Where appropriate, information was presented visually to increase general understanding and accessibility for women with lower levels of health literacy.

#### Participant booklets

**Fig. 3:**
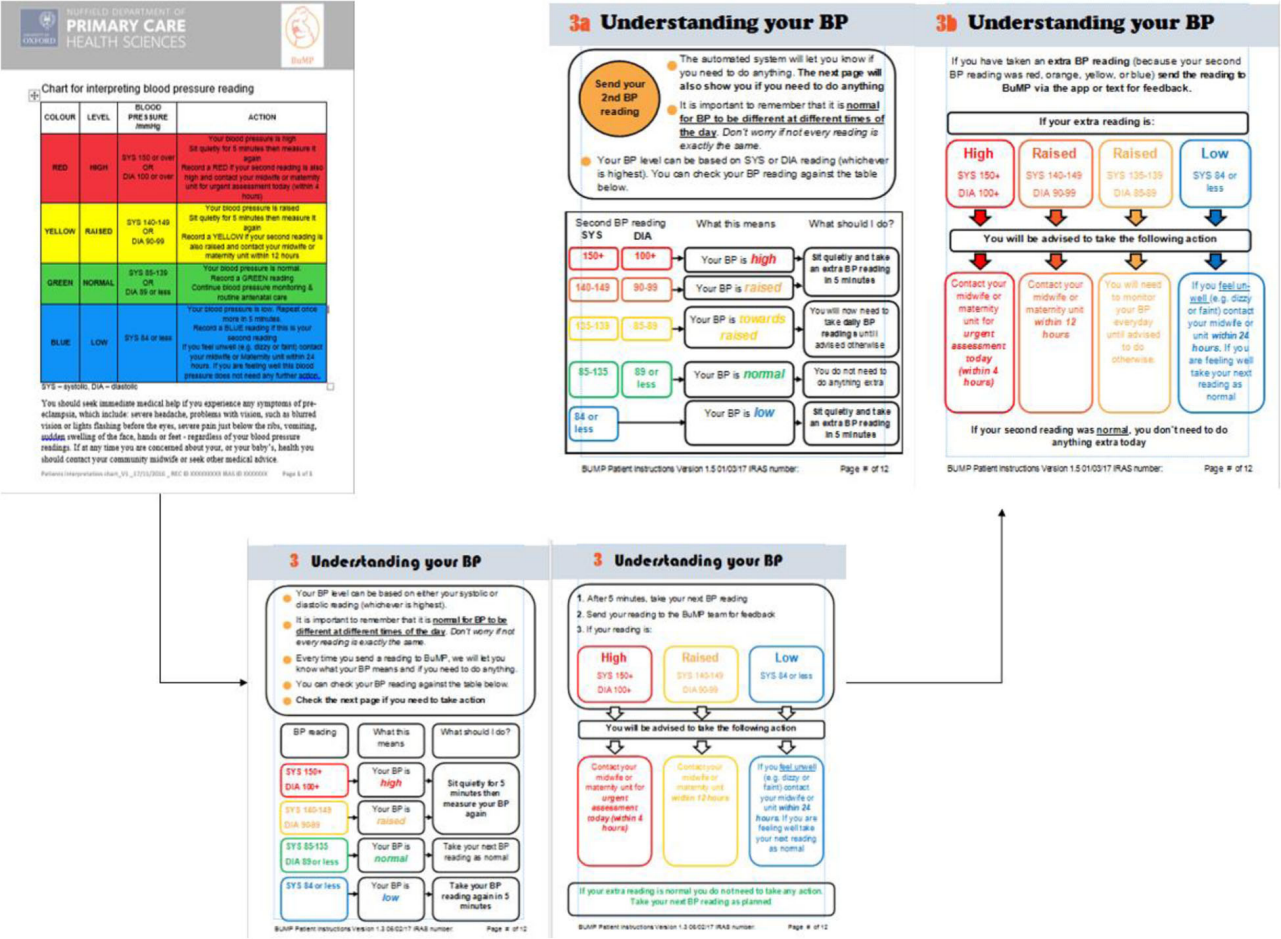
llustration of the iterative refinement of the BP feedback information given to intervention participants (note: Version 1 is the leftmost version presented below and final version is rightmost in figure)

The participant booklet incorporated several previous documents outlining instructions for BP monitoring, BP interpretation charts and the telemonitoring specification document. Each version was reviewed by the development group and target users, who provided feedback on aspects that were particularly salient, but crucially, aspects that were off-putting or difficult to understand [18]. The information in the previous documents was checked for reading age and went through ten iterations to ensure that it was as clear and as simple as possible. For example, participants queried the BP reading feedback and highlighted where there were inconsistencies from the user perspective (such as using “last reading” and “extra reading” to describe the same thing). In relation to the actions described for “normal” BP readings, users suggested that we used “today” when advising that no further action was necessary. Additional sections addressing the rationale for checking BP were also included: information about pre-eclampsia, outlining the full range of symptoms to be aware of in addition to high BP; information about BP variability; habit formation; and overcoming barriers around missed readings. Figure 3 illustrates some of the iterative changes made to the patient booklet.

#### Participant information sheets

As the key challenge here was to ensure that all women could easily understand what participation involved, it was important that the information provided was not too overwhelming and wordy that it might be off-putting. Consequently, the PIS was modified from four A4 pages to a four-page, A5 booklet format, ordering the most important information first. This process included nine iterations. A flowchart was developed and modifications made as a result of user feedback, for example, changing “after delivery” to “after birth”, and eliciting feedback to identify the most comprehensible way to explain the monitoring regime (described as “This will mean taking 2 readings, 5 minutes apart (10 minutes total) on at least 3 days each week”).

#### Text messages

Motivational text messages were developed specifically targeting key behavioural issues to be delivered to intervention group participants. Message length was kept to a minimum (i.e. approximately the length of one SMS message) and content checked for readability (aiming for an average reading age of 10–11 years, where possible). After the first iteration and in consultation with the expert development team, it was decided that women would receive one message selected at random, apart from the first week, when they would be congratulated for taking part in the study. Previous work highlighted that addressing women by name was important in increasing engagement with text messages in interventions aimed at pregnant women [52], and this was also included in the BUMP text messages. The development of the text messages was organised around the themes emerging from the planning process. For example, messages reinforced beliefs that self-monitoring may be helpful for addressing general worries about health and can be undertaken flexibly at home and in response to feeling unwell. We suggested strategies to overcome likely difficulties (such as ways to deal with transitional periods which disrupt routines) by addressing environmental factors that can facilitate (or act as a barrier) to the successful enactment of self-monitoring. Specific wording of messages was checked with the women participating in the iterative qualitative feedback. Fifty-five text messages were developed arranged in ten categories. These are outlined, alongside an example message in Table 3. The messages are randomly selected from a pool of messages, dependent on the woman’s phase in the study.

**Table 3:** Examples of text messages developed within each category

Category	Example
Congratulating for taking part	Hi [Firstname]. It’s great that you have signed up to BUMP. Taking your readings at home is an excellent way to learn more about your BP – you can also track it over time using the website or the app.
Health benefits of self-monitoring	Hi [Firstname]. Some women find that taking their own BP helps them to notice changes more quickly than they would normally. Log in now or text to send your reading.
Reassurance about the safety of taking part in the study	Hi [Firstname]. The best thing about checking your own BP is knowing when your BP is higher than normal. When this happens we will help you take action to manage it! Log in to find out more.
Risks associated with high BP/pre-eclampsia	Hi [Firstname]! Did you know that high BP affects about 1 in 10 women during pregnancy? Checking at home can help you quickly notice if your BP is too high!
Habit formation	Hi [Firstname]. A great way to get in the habit of taking your BP is to choose a time to suit you and setting an alarm on your phone as a reminder!
Keeping on track	Hi [Firstname]. It can be tricky to remember to take your BP! Using the BUMP app or website can help keep you on track and let you know what to do if your BP is too high! Why do not you log in today?
Information about BP variability/changes	Hi [Firstname]. Did you know that BP can change day-to-day and at different times of day? The great thing about checking at home is that we will have lots of readings to base any decisions about your care!
Risk in later pregnancy	Hi [Firstname]. BP often rises in the last few weeks of pregnancy – knowing what’s normal for you will help you notice if it starts to rise! You can see all your readings in the app or online.
Setbacks/missed readings (reassurance/what to do)	Hi [Firstname]. The odd missed reading does not matter. Do not worry, as long as you take a reading as soon as you can! Text or log in to send your reading today.
Disruption/changes to routine	Hi [Firstname]. Making a plan can help when it’s hard to remember to take your BP. Why not try keeping the monitor somewhere to remind you in the morning?

### The BUMP logic model

**Fig. 4:**
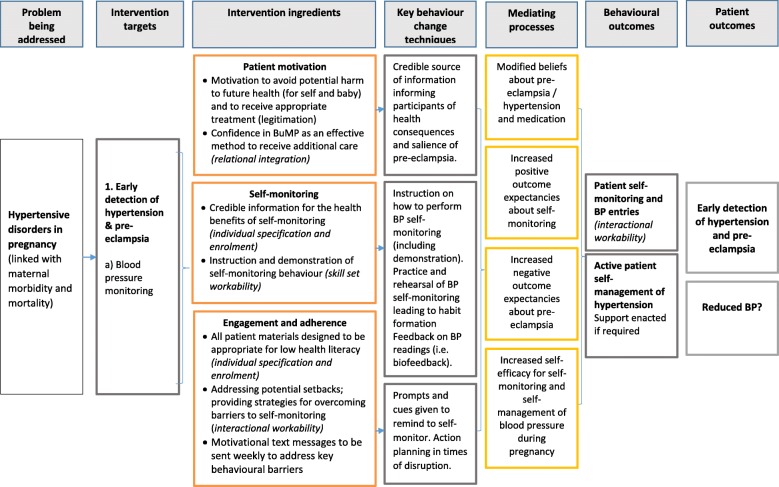
The BUMP logic model

The BUMP logic model is presented in Fig. 4. It was hypothesised that the intervention would affect a number of mediating processes through which participant outcomes would be influenced. Based on the planning process, we proposed that the intervention would modify women’s beliefs about hypertension, pre-eclampsia and its treatment, specifically by increasing positive outcome expectancies (i.e. the perceived benefits) about self-monitoring and negative outcome expectancies (i.e. harmful consequences) of pre-eclampsia. In addition, increased self-efficacy for SMBP during pregnancy was anticipated to be the key mediating process [53]. All mediating processes were hypothesised to directly impact on the target behavioural outcomes. Accordingly, items regarding self-efficacy for SMBP, beliefs about blood pressure and beliefs about a medication (taken from the beliefs about medication questionnaire (BMQ)) [53] were included in baseline and follow-up assessments to facilitate confirmation of these proposed processes.

The primary outcome of the BUMP1 trial will be the early detection of hypertension and pre-eclampsia compared with usual care. However, there are other mediating behavioural outcomes which directly influence the extent to which the primary outcomes are likely to occur. These involve participants undertaking SMBP, entering these measurements into the app (or telemonitoring system), and then actively engaging with the automated feedback provided, specifically when further action is needed for readings above target.

## Discussion

This paper has described the process of developing and modifying the BUMP pilot intervention which aims to facilitate the early detection of hypertension (and subsequently pre-eclampsia) in pregnant women using evidence, theory and the person-based approach to ensure that behavioural issues were addressed ahead of the BUMP1 RCT. Despite best practice advice (such as that outlined within MRC guidance for developing and evaluating complex interventions) [51, 54], in-depth development work aimed at promoting acceptability and engagement for a wide spectrum of target users remains under-developed (or at least under-reported) in practice [14]. This is especially true for digital interventions aimed at pregnant women: although there are many available, very few have been explicitly developed using rigorous scientific approaches (i.e. theory or evidence based), alongside in-depth acceptability testing, or tested using gold-standard methods such as RCTs [18, 55].

The intervention planning and development guided by a person-based approach ensured the novelty of the BUMP interventions by incorporating complementary theory, evidence and person-based approaches. By using these methods in a coherent way, several key insights informed design modifications to enhance the potential acceptability of the intervention and engagement with SMBP throughout pregnancy. This process helped the research team to develop a deep appreciation of the issues women report around their experiences of hypertensive disorders in pregnancy, particularly in relation to how they understand the risks and consequences associated with pre-eclampsia and how SMBP is relevant in detecting this. By identifying women’s difficulties in making sense of pre-eclampsia and its implications for their health and the health of their baby, we were able to directly address these issues within the participant booklet and text messages. Guiding principles facilitated the decision-making process throughout by maintaining the core design objectives and key features, for example, by ensuring that all information was presented in a clear but simple way [18]. In addition, exploration of the practicalities of incorporating self-monitoring into daily life during pregnancy allowed a targeted approach to address common barriers (such as providing strategies to overcome disruptions or major changes in routines), alongside providing women with information about the potential health benefits of self-monitoring. Including iterative qualitative work throughout the development process ensured that the intervention materials were engaging and acceptable to women, before implementation in a full-scale RCT.

The theoretical modelling undertaken as part of developing the logic model provided an overview of the hypothesised causal mechanisms of change and, in doing so, informed the inclusion of behavioural items within the process evaluation, in line with the best practice guidelines by the MRC [51]. In addition, the documentation of the planning and development process complements other such accounts of similar processes focused on self-monitoring more broadly [16]. The BUMP trials are the first adequately powered studies to assess the impact of self-monitoring of blood pressure in pregnancy [11]. This study builds on accumulating evidence that digital interventions are effective in reducing BP compared with usual care in a general population [56]. Recruitment for the BUMP trials ended in September 2019. Over 3000 women were recruited overall (2441 to BUMP1 and 600 to BUMP2) this was above our initial target recruitment and took place within the planned recruitment time. The follow-up period for these trials will continue until spring 2020.

The intervention development described here illustrates that it is possible to integrate several methods to elicit the issues surrounding interventions specifically aimed at pregnant women and that it is feasible to address barriers to behaviour change within the intervention and participant materials. Using the PBA [18] aimed to ensure the intervention was engaging, persuasive and acceptable by working from an in-depth understanding of the target user. While we were able to explore the views and reactions of target users to the BUMP1 materials, none of the women were able to actually undertake SMBP or use the telemonitoring system within this specific intervention development phase. The qualitative work that will be embedded within the main BUMP trials will seek to explore the success of integrating the key target behaviours in actual practice.

## Conclusions

This development work has aimed to address the known barriers and facilitators within the intervention, resulting in an intervention that is fit for testing. The BUMP trials will assess the extent to which these interventions can facilitate the early detection and management of hypertension in pregnancy.

## Data Availability

The data that support the findings of this study are available from the University of Oxford but restrictions apply to the availability of these data, which were used under licence for the current study, and so are not publicly available. Data are however available from the authors upon reasonable request and with permission of the University of Oxford.
